# Insight into the Morphological Properties of Nano-Kaolinite (Nanoscrolls and Nanosheets) on Its Qualification as Delivery Structure of Oxaliplatin: Loading, Release, and Kinetic Studies

**DOI:** 10.3390/molecules28135158

**Published:** 2023-07-01

**Authors:** Mashael Daghash Alqahtani, Nourhan Nasser, May N. Bin Jumah, Saleha A. AlZahrani, Ahmed A. Allam, Mostafa R. Abukhadra, Stefano Bellucci

**Affiliations:** 1 Department of Biology, College of Science, Princess Nourah bint Abdulrahman University, P.O. Box 84428, Riyadh 11671, Saudi Arabia; mdalqahtani@pnu.edu.sa (M.D.A.);; 2 Geology Department, Faculty of Science, Beni-Suef University, Beni-Suef 65211, Egypt; 3 Materials Technologies and Their Applications Lab, Geology Department, Faculty of Science, Beni-Suef University, Beni-Suef 65211, Egypt; 4 Zoology Department, Faculty of Science, Beni-Suef University, Beni-Suef 65211, Egypt; ahmed.aliahmed@science.bsu.edu.eg; 5 INFN-Laboratori Nazionali di Frascati, Via E. Fermi 54, 00044 Frascati, Italy

**Keywords:** kaolinite, exfoliation, methanol, oxaliplatin, loading, cytotoxicity

## Abstract

Natural kaolinite underwent advanced morphological-modification processes that involved exfoliation of its layers into separated single nanosheets (KNs) and scrolled nanoparticles as nanotubes (KNTs). Synthetic nanostructures have been characterized as advanced and effective oxaliplatin-medication (OXAP) delivery systems. The morphological-transformation processes resulted in a remarkable enhancement in the loading capacity to 304.9 mg/g (KNs) and 473 mg/g (KNTs) instead of 29.6 mg/g for raw kaolinite. The loading reactions that occurred by KNs and KNTs displayed classic pseudo-first-order kinetics (R^2^ > 0.90) and conventional Langmuir isotherms (R^2^ = 0.99). KNTs exhibit a higher active site density (80.8 mg/g) in comparison to KNs (66.3 mg/g) and raw kaolinite (6.5 mg/g). Furthermore, compared to KNs and raw kaolinite, each site on the surface of KNTs may hold up to six molecules of OXAP (*n* = 5.8), in comparison with five molecules for KNs. This was accomplished by multi-molecular processes, including physical mechanisms considering both the Gaussian energy (<8 KJ/mol) and the loading energy (<40 KJ/mol). The release activity of OXAP from KNs and KNTs exhibits continuous and regulated profiles up to 100 h, either by KNs or KNTs, with substantially faster characteristics for KNTs. Based on the release kinetic investigations, the release processes have non-Fickian transport-release features, indicating cooperative-diffusion and erosion-release mechanisms. The synthesized structures have a significant cytotoxicity impact on HCT-116 cancer cell lines (KNs (71.4% cell viability and 143.6 g/mL IC-50); KNTs (11.3% cell viability and 114.3 g/mL IC-50). Additionally, these carriers dramatically increase OXAP’s cytotoxicity (2.04% cell viability, 15.4 g/mL IC-50 (OXAP/KNs); 0.6% cell viability, 4.5 g/mL IC-50 (OXAP/KNTs)).

## 1. Introduction

Noncontagious diseases, particularly the most frequent form of cancer, have been responsible for the vast majority of fatalities worldwide, and this influence is expected to increase by 75% in the next few years [1,2]. Colorectal cancer, one of the most frequently occurring cancers of the digestive system, affects roughly 13% of cancer patients worldwide [3,4]. It has a significant detrimental effect on human life and is one of the two primary top variables that result in death and increase global rates of mortality. In the mucosal layers, colorectal malignancy initially appeared as a polyp before spreading to the submucosa and adjacent tissues. Then, during its most advanced phases, the oncologic cells extensively infiltrated the lymph nodes as well as adjacent organs [5,6,7]. To inhibit the ongoing and continuing spread of cancer cells, a variety of chemotherapy treatments have been implemented [8,9]. However, the majority of frequently applied chemotherapies are toxic to normal fresh cells as well as having a number of adverse effects on a variety of organisms, such as kidney damage and bone-marrow suppression [4,5]. To enhance the selectivity as well as the biological safety of the most commonly utilized chemotherapies, several strategies have been investigated [5]. This entailed developing innovative forms of chemotherapy or improving the therapeutic and safety attributes of widely used medications to meet global demand and the expense of living in developing and underdeveloped nations [3].

Oxaliplatin (OXAP) has been confirmed as one of the most successful chemotherapy drugs employed for the therapy of cancerous cells owing to its ability to produce active platinum-based structures that have an intense inhibitory effect on the replication of DNA in tumor cells [3,4,10]. However, the OXAP drug was approved by the FAD organization to be applied as chemotherapy during the treatment of the metastatic stages of cancer, and its metabolic byproducts and associated derivatives demonstrate considerable toxic effects on healthy and infected cells [7,11]. OXAP revealed a variety of serious adverse effects during the course of therapy, including myelotoxicity, cardiotoxicity, and gastrointestinal problems [7,12]. Furthermore, additional concerns connected to OXAP’s poor bloodstream solubility have been reported [6,13]. As a result, a variety of innovative delivery techniques were developed as a successful approach for enhancing the selectivity, solubility, curative value, release rate, and therapeutic effects of OXAP drugs and can also maintain the provided dose of the medication at the recommended quantities [11,12]. This may minimize the adverse impacts of the medication on other healthy cells and regulate how long carcinogenic cells remain exposed to the medication ions [9,11].

Several types of inorganic, organic, and organic/inorganic hybrid structures have been described as successful delivery vehicles for the OXAP drug in addition to various forms of chemotherapies [9,14,15]. These structures significantly enhance the retention and permeation impacts of anticancer medications. Alginate, cellulose/zeolite, cyclodextrin/phillipsite, mesoporous silica, bentonite/cellulose, polymers, lipid nanoparticles, and liposomes were among the materials that were used in these formulations [4,9,11,12]. Clay minerals, such as kaolinite, vermiculite, montmorillonite, sepiolite, and halloysite, were widely reported as the most effective carriers of the common chemotherapies. The majority of clay minerals have distinct layered aluminosilicate frameworks that have significant ion-exchange capability, biological compatibility, non-toxic nature, stable chemical properties, adsorption capacity, affordable prices, thermal resistance, and flexible chemical properties [16,17,18,19].

Kaolinite clay mineral is a naturally occurring hydrous aluminum silicate material with a 1:1 intercalated tetrahedron/octahedron framework [12,20]. Despite the fact that the kaolinite mineral is abundant in nature and inexpensive in comparison with frequently encountered industrial clay minerals such as montmorillonite, the studies introduced on its potential as a therapeutic delivery structure remain insufficient [12,21]. This was attributed to the estimated small surface area, quick release rate, weak ion-exchange capability, and poor capacity to absorb drugs in contrast to bentonite and halloysite, which are the two most extensively utilized clay-based carriers of drugs [22]. Consequently, various modification methods have been implemented to enhance the physical and chemical qualities of kaolinite, including scrolling, exfoliation, organic functionalization (organosilanes and amino alcohols), polymeric intercalation, and inorganic hybridization.

The morphological properties of the prepared materials have a significant influence on their chemical, biological, and physical properties, as the morphology can significantly influence the adsorption capacity, surface area, and extent of active site exposure [23]. Producing nanomaterials with one-dimensional (nanotubes and nanorods) or two-dimensional geometries has been proposed for many applications as a result of their remarkable surface area, excellent dispersion properties, and significant surface reactivity [24,25,26]. In more recent years, the exfoliation of the layered units of clay minerals into separated forms of single silicate sheets with two-dimensional forms has been developed as a highly advanced modification methodology. This technique was successfully applied to produce innovative nanostructures of clay minerals that have significant biological compatibility, adsorption capacity, oxidation characteristics, surface reactivity, anticancer activity, surface area, and dispersion properties [12,27]. However, this method was extensively addressed for montmorillonite, while only a few studies concerning exfoliated kaolinite have been described [12,27]. Additionally, one-dimensional nanostructures with notable high biological activity, chemical reactivity, surface area, and catalytic performances were suggested as highly advanced nanomaterials for a variety of applications [12,25,26]. Synthetic kaolinite nanoscrolls or nanotubes have recently been recognized as innovative and efficient adsorbent materials with outstanding surface area, an advanced porous framework, and significant reactivity [28]. By simply exfoliating kaolinite sheets, dealumination transforms them into nanoscrolls under the influence of ultrasonic sound waves and chemical-expansion reactions. The resulting material has semicrystalline characteristics, a significant surface area, a well-ordered porous framework, and an outstanding adsorption capacity [22].

Unfortunately, the impact of the morphology of the kaolinite nanostructures on its properties and biological activity as a drug-delivery system and anticancer agent has not been covered by satisfactory studies yet. Therefore, the presented study involved deep investigation for the qualification of synthetic kaolinite nanosheets and kaolinite nanoscrolls as nano-delivery structures of the oxaliplatin drug during the treatment of colorectal cancer. The study involved a detailed assessment of the loading properties, release profiles, equilibrium, and thermodynamics of the loading properties, release kinetics, and cytotoxicity studies.

## 2. Results and Discussion

### 2.1. Characterization of the Carrier

**Figure 1:**
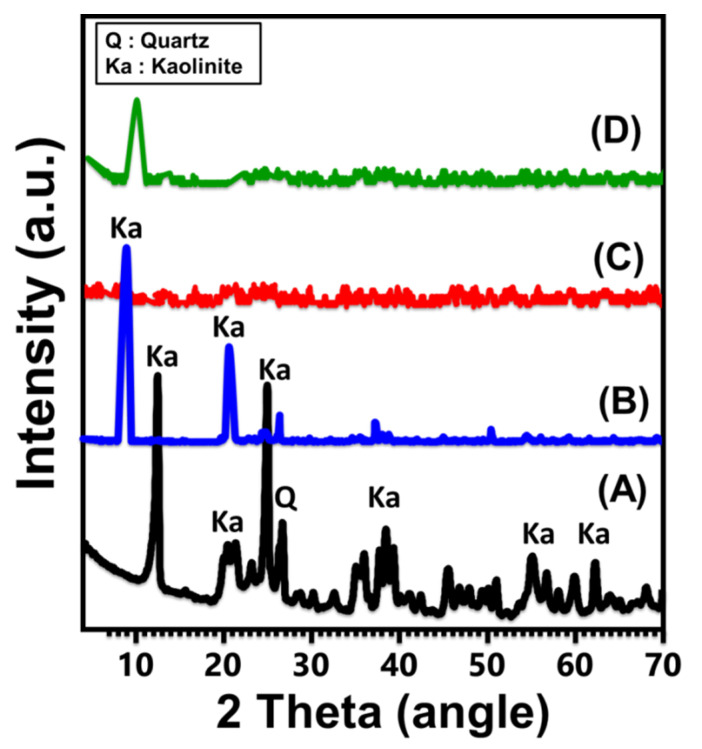
XRD patterns of raw kaolinite (**A**), DMSO-modified kaolinite (**B**), exfoliated kaolinite sheets (**C**), and the synthetic kaolinite nanotubes (**D**).

Based on the XRD patterns, structural transformations from the crystalline properties of kaolinite raw minerals to single nano-kaolinite sheets (KNs) and kaolinite nanotubes (KNTs) were monitored. The initial phase of kaolinite displays normal peaks (12.33° (001), 20.85° (−110), 24.87° (002), and 26.64° (111)) of triclinic, highly crystallized kaolinite with its d-spacing value (0.72 nm). After the DMSO intercalation step, the majority of the kaolinite characteristic diffraction peaks were significantly diminished, with the exception of the matching peaks (001) and (002), which were notably deviated (Figure 1B). The diffraction pattern that was obtained after the sonication-induced CTAB exfoliation process (KNs) demonstrated a full diminution for all remaining peaks, and the modified material appeared to display an amorphous crystalline structure (Figure 1C). This confirms that the kaolinite layers were successfully split into independent, single silicate layers of nano-crystalline or semi-crystalline nature. The synthetic kaolinite nanotubes, or scrolled kaolinite (KNTs), also display an XRD pattern with a noticeable reduction for the kanon peaks of kaolinite and the existence of a new reduced peak at a 2 Theta angle of about 10.6°, which is the significant peak of the (001) crystallographic planes of scrolled kaolinite (Figure 1D).

**Figure 2:**
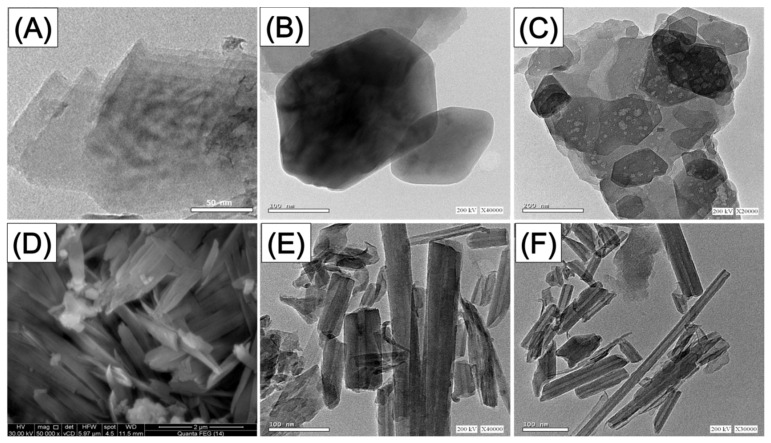
HRTEM images of synthetic exfoliated kaolinite particles (**A**–**C**), SEM image of synthetic kaolinite nanotubes (**D**), and the HRTEM images of the synthetic kaolinite nanotubes (**E**,**F**).

Regarding the morphological transformation during the different synthesis procedures, the starting kaolinite grains appeared as stacked pseudo-hexagonal flaky or platey-like particles either in the recognized SEM images (Figure S1A) or the HRTEM images (Figure S1B). The exfoliated products’ acquired HRTEM images show that the kaolinite has been significantly stripped away and separated into single layers (Figure 2A). Other analyzed images revealed the presence of the separated kaolinite silicate sheets with notable preservation of the pseudo-hexagonal outline but with smoother margins than the raw kaolinite flakes (Figure 2B). Some samples have lighter gray tones in contrast with the kaolinite sheets’ overall gray tone, revealing disorder in the structural silicate units of kaolinite (Figure 2C). The successful production of the KNTs has also been verified by SEM and HRTEM pictures (Figure 2D–F). The kaolinite mineral’s flakes were subjected to a significant transformation into curled or scrolled nanoparticles with tabular shapes (Figure 2D). The scrolling particles were found to be tubes with tubular hollow patterns with interior diameters ranging from 2 to 20 nm (Figure 2E,F). The length of the developed KNTs ranged from almost 50 nm to over 600 nm, and the external diameter was found to be between 10 nm and 50 nm. The morphological transformation of kaolinite is associated with remarkable enhancement in the determined surface area as the value of raw kaolinite (10 m^2^/g) increased to 80.2 m^2^/g and 105 m^2^/g after the exfoliation (KNs) and scrolling processes (KNTs), respectively.

The changes in the particle-size distribution of the addressed carriers before and after the loading processes were measured using a laser diffraction particle-size analyzer. The main particle diameter of free kaolinite is 86 ± 3 µm while the OXAP-loaded particles have a slightly higher mean diameter (89.5 µm). The same observations were detected after the loading of both KNs and KNTs with the drug molecules. The mean particle diameter of KNs increased from 630 nm to 683 nm while the determined mean value of KNTs increased slightly from 312 nm up to 336 nm.

**Figure 3:**
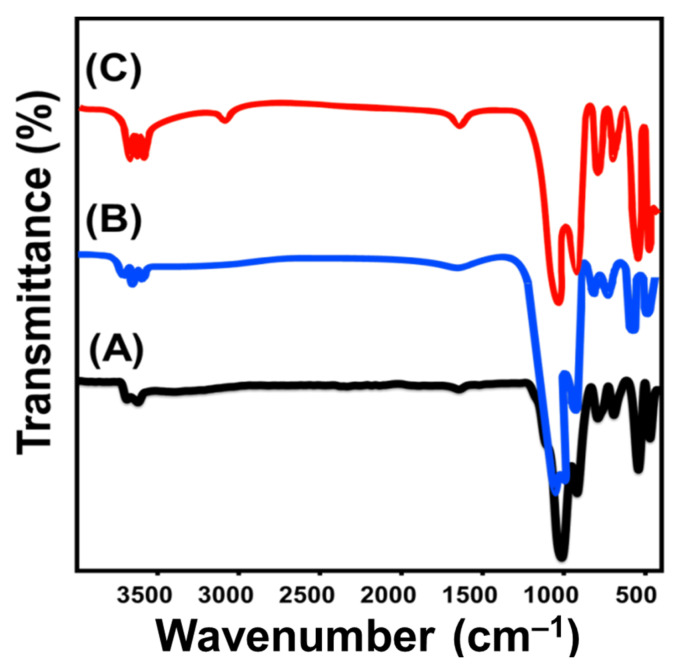
FT-IR spectra of raw kaolinite (**A**), exfoliated kaolinite sheets (**B**) and the synthetic kaolinite nanotubes (**C**).

Regarding the chemical properties, the impact of the morphological-transformation process from kaolinite into KNs and KNTs on the structural chemical groups was assessed according to their FT-IR spectra. The spectrum of kaolinite displays clearly the characteristic groups of its aluminosilicate structure, including Si-O (787 and 456 cm^−1^), Si-O-Al (526 and 680 cm^−1^), Si-O-Si (1020 cm^−1^), Al-OH (912 and 3500 cm^−1^), O-H (1641 cm^−1^), and Si-OH (3689 cm^−1^) [20,29] (Figure 3A). The detected spectrum of KNs shows exactly the same absorption bands corresponding to those observed in raw kaolinite, but with substantial shifts in their positions, reductions in their intensity, and splitting of identifiable bands at about 900 cm^−1^ and 1000 cm^−1^ (Figure 3B). This denotes effective exfoliation of the aluminosilicate layers of kaolinite into monolayer layers or separate sheets and predicted distortions of its octahedron and tetrahedron units [22,30] (Figure 3B).

The same observations were reported during the investigation of the FT-IR spectrum of the synthetic KNTs particles (Figure 3C). The corresponding bands of the aluminosilicate structure deviated significantly as compared to their positions in kaolinite. This also signifies the distortion effect of the exfoliation and scrolling modification on the structural octahedron and tetrahedron units of kaolinite, in addition to the impact of the newly formed hydrogen bonds between the used organic reagents (CTAB and methanol) and the hydroxyl-bearing functional groups of kaolinite (Figure 3C) [22]. After the loading process of OXAP into K, KNs, and KNTs, the resulting FT-IR spectra demonstrate the successful loading of the drug molecules. The interaction between the drug ions and the active chemical groups of K, KNs, and KNTs resulted in a considerable deviation in the positions of the corresponding bands of the essential functional groups. Moreover, the loaded OXAP drug was confirmed based on the detected bands of its chemical structure, such as the asymmetric and symmetric Pt–O binding (K (831.3 cm^−1^ and 1297.3 cm^−1^) (Figure S2A), KNs (826.7 cm^−1^ and 1290 cm^−1^) (Figure S2B), and KNTs (818.7 cm^−1^ and 1289.2 cm^−1^) (Figure S2C) [12,31,32].

### 2.2. Encapsulation of OXAP Drug

#### 2.2.1. Influence of the Encapsulation Parameters

##### Effect of pH

**Figure 4:**
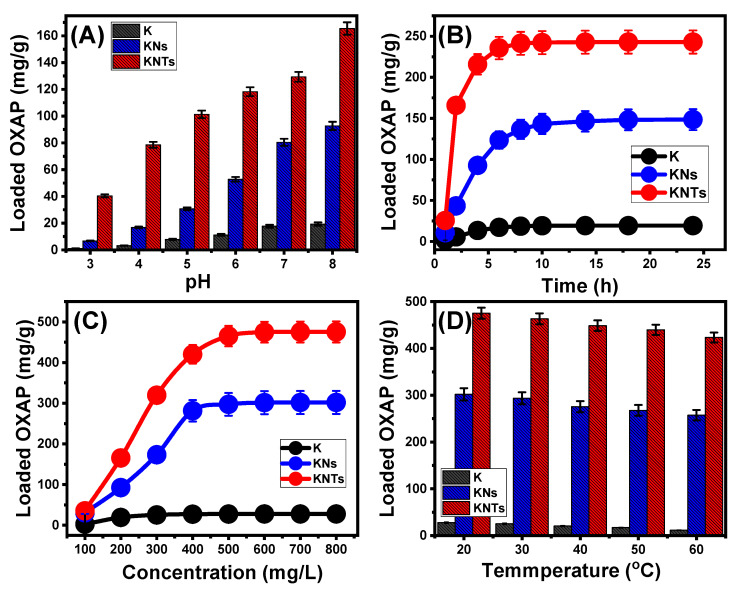
Effect of the experimental variables on the loading of OXAP into K, KNs, and KNTs including the pH (**A**), loading duration (**B**), OXAP concentration (**C**), and loading temperature (**D**) (*p* < 0.05; *n* = 3).

The pH of the solution being used has a significant impact on the encapsulating effectiveness of OXAP into K, KNs, and KNTs by directing both the ionization nature of the drug and the dominant charges on the carriers’ surfaces. Following specific parameters (25 mg (carrier dose), 200 mg/L (drug concentration), 50 mL (volume), 4 h (duration), and 20 °C (temperature)), the influence of pH was monitored from pH 3 to pH 8. Experimentally, the OXAP-loading capacities of K, KNs, and KNTs rise considerably with increasing pH from pH 2 (1.1 mg/g (K), 6.7 mg/g (KNs), and 40.4 mg/g (KNTs) towards pH 8 (12.4 mg/g (K), 92.7 mg/g (KNs), and 165.5 mg/g (KNTs) (Figure 4A). This behavior was attributed to an increase in both the solubility and mobility behaviors of OXAP in solutions with an acidic pH [4,15]. Additionally, [Pt(dach)(H_2_O)Cl]^+^ and [Pt(dach)(H_2_O)_2_]^2+^ are the most predominant and stable ionized forms produced whenever the OXAP drug is dissolved at such acidic pH levels [16]. These OXAP ions that are positively charged exhibit a high electrostatic repulsion nature with the protonated chemical structures of K, KNs, and KNTs, which are saturated with numerous positive hydronium ions [33]. As a result, the basic state is favored throughout the loading of OXAP into KNs and KNTs, which is consistent with the measured pH_(PZC)_ values of KNs (pH = 6.8) and KNTs (pH = 6.4).

##### Encapsulation Interval

The encapsulation characteristics of K, KNs, and KNTs with regard to loading duration are essential factors in determining the equilibrium interval of the OXAP loading and controlling the loaded quantity in accordance with the proposed dosage. The influence of encapsulation periods was monitored from 1 h to 24 h, whereas other impacting experimental variables were kept constant (25 mg (carrier dose), pH 8, 50 mL (volume), 20 °C (temperature), and 200 mg/L (OXAP concentration)). The OXAP encapsulation capacities of K, KNs, and KNTs demonstrate an adequate enhancing effect for the rise in the duration of the tests up to 10 h for K, 18 h for KNs, and 8 h for KNTs (Figure 4B). Following that, the experimental loading rate shows no significant changes or is almost stable, and there is no notable increase in the quantity of encapsulated OXAP, establishing the equilibrium states of the carriers (19.3 mg/g (K), 148.5 mg/g (KNs), and 243 mg/g (KNTs)) (Figure 4B). The number of active and free sites that were present on the surfaces of K, KNs, and KNTs at the beginning of the experiments led to a noticeably higher actual encapsulation rate of OXAP [28]. As more sites are occupied with additional OXAP as the loading period is extended, the number of accessible sites gradually diminishes, leading to a reduction in the rate of the reaction. The full occupation of such active sites led to an equilibrium setting, where no additional OXAP molecules could be loaded [34].

##### OXAP Concentration

The maximal encapsulating capacities, equilibrium characteristics, and regulation of the loaded OXAP dosage all depend on the encapsulation behaviors of K, KNs, and KNTs in relation to the studied OXAP concentration. Following specific values of the affecting variables (25 mg (carrier dose), pH 8, 50 mL (solution volume), 20 °C (temperature), and 24 h (duration)), the influence of OXAP concentrations was monitored from 100 to 800 mg/L. The high OXAP concentrations have a favorable impact on the observed encapsulation characteristics of K, KNs, and KNTs (Figure 4C). OXAP ions’ driving forces and diffusion properties are induced by the presence of high quantities of OXAP ions, which improves their chances to interact with effectiveness encapsulation sites and, consequently, the OXAP-loading capacities of K, KNs, and KNTs [35,36]. This enhancement impact was observed up to an OXAP concentration of 500 mg/L for K and KNs, and 600 mg/L for KNTs. Following that, the increase in OXAP concentration showed a neglect effect reflecting an equilibration state (Figure 4C). As a result, these concentrations (500 mg/L for K and KNs, and 600 mg/L for KNTs) demonstrate the saturation concentrations of K, KN, and KNTs at which they reach their experimental highest loading capacities (27.7 mg/g for K, 302 mg/g for KNs, and 475.3 mg/g for KNTs) (Figure 4C). The observable higher OXAP encapsulation capacities of KNs and KNTs can be attributed to the significant enhancement in the surface area as well as the reactivity of the aluminosilicate sheets that appear with semi-crystalline properties and high exposed active sites, especially the siloxane groups.

##### Effect of Temperature

The drug-loading assays were conducted with a gradual increase in temperature from 20 to 60 °C to determine if it affected K, KNs, and KNTs’ OXAP-loading capacities in a favorable or unfavorable manner (Figure 4D). All the experimental variables of the loading process were adjusted at 24 h (loading period), 800 mg/L (OXAP concentration), 50 mL (the solution volume), pH 8, 20 mg (carrier dosage), and 20 °C (temperature). The decrease in the OXAP-loaded quantities as the investigated loading temperature rises (Figure 4D) supports the exothermic nature of K, KNs, and KNTs’ loading mechanisms. At 60 °C, K, KNs, and KNTs have loading capacities of 11.8 mg/g, 257.4 mg/g, and 423.4 mg/g, respectively (Figure 4D). According to the findings of loading studies, both KNs and KNTs demonstrate attractive qualities as OXAP carriers due to their high loading capacities. Furthermore, by adjusting several loading variables, such as pH, duration, drug concentration, and temperature, it is possible to manage the quantity of the entrapped drug on both KNs and KNTs.

#### 2.2.2. Encapsulation Mechanism

##### Kinetic Properties

Intra-Particle Diffusion Behavior

**Figure 5:**
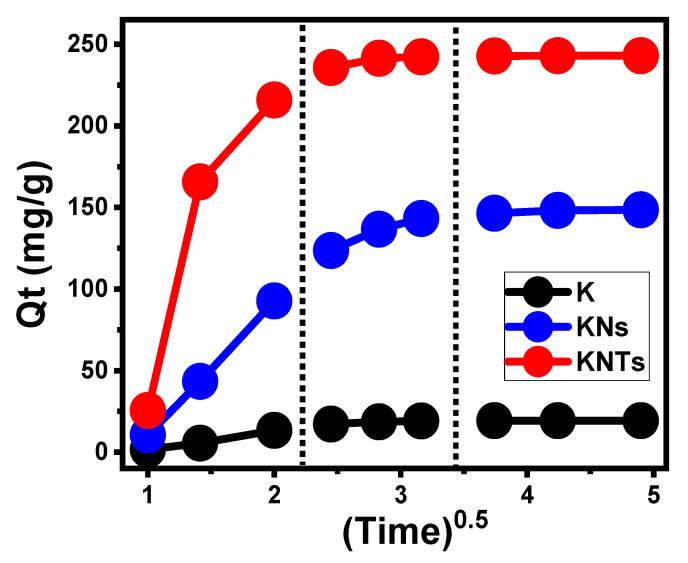
The intra-particle diffusion curves of the loading process of OXAP into K, KNs, and KNTs.

Intra-particle diffusion curves with three distinct stages and no crossovers with the initial points of the curves have been detected for the loading reactions of OXAP into K, KNs, and KNTs (Figure 5). This reveals the encapsulation of OXAP by collaborative mechanisms in conjunction with the substantial impact of the diffusion of ions towards the active receptors of K, KNs, and KNTs [35,37]. This could involve (A) loading by the distributed active sites over the exterior surface (border), (B) intra-particle diffusion, and (C) the mechanistic impact of the equilibrium stages [38]. The occurrence of the first stage denotes the activity of the exterior encapsulation mechanisms during the initial stages of the experiments, and the quantity of the surface-active receptors controls how effectively the encapsulation reactions proceed (Figure 5) [39]. By extending the encapsulation period, a new stage has been observed (Figure 5) that denotes the existence of additional mechanisms, including the impact of the OXAP-diffusion actions and the layered encapsulation activities. Finally, the equilibrium states of K, KNs, and KNTs during the OXAP-loading reactions show the third stage to be the most predominant stage. This implies that the encapsulated OXAP ions have occupied or consumed all the efficient binding sites (Figure 5) [5,36]. During this step, multiple mechanisms influence the encapsulation reactions, which may involve molecular interaction as well as an interionic attraction [35].

Kinetic Modeling

**Figure 6:**
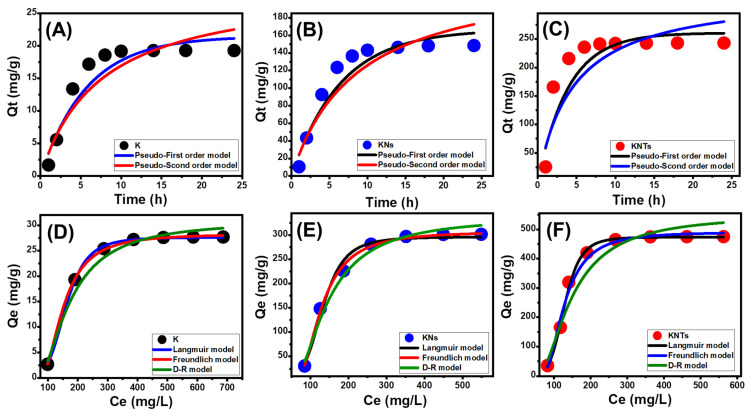
Fitting of the OXAP-loading results with the different kinetic models (raw kaolinite (**A**), synthetic KNs (**B**), and synthetic KNTs (**C**)) and the classic equilibrium models (raw kaolinite (**D**), synthetic KNs (**E**), and synthetic KNTs (**F**)).

The kinetic characteristics of the pseudo-first-order (PFO) (Equation (1) and pseudo-second-order (PSO) (Equation (2) models were used to evaluate the kinetic properties of the OXAP-encapsulation processes performed by K, KNs, and KNTs. This had been completed by non-linearly fitting the results with the models’ illustrative equations while considering the correlation coefficient (R^2^) and chi-squared (χ^2^) values as indicators of the fitting degree (Table 1, Figure 6A–C).

(1) $$Q_{t\;}=Q_{e}\;\left(1-e^{-k_{1}.t}\right)$$

(2) $$Q_{t}=\frac{Q_{e\;}^{2}k_{2}t}{1+Q_{e}k_{2}t}$$

According to the established values of R^2^ and χ^2^, OXAP was encapsulated into K, KNs, and KNTs in agreement with the kinetic specifications of the PFO model as compared to the kinetic characteristics of the PSO model (Table 1). This was further substantiated by the observation that the calculated OXAP-loading equilibrium capacities as parameters of the PFO model (21.47 mg/g (K), 166.6 mg/g (KNs), and 250.7 mg/g (KNTs) were close to the actually obtained values (19.3 mg/g (K), 148.5 mg/g (KNs), and 243 mg/g (KNTs). Such kinetic features signify the existence of physical OXAP-loading mechanisms, which might include electrostatic attractions [40,41]. However, the observed notable fit of the performed OXAP-loading reactions by K, KNs, and KNTs with PSO kinetics at acceptable degrees suggests a considerable impact of some weak chemical processes as essential or as assistance mechanisms. This might include weak chemical interactions, such as electron exchanges, hydrogen bonding, and electron sharing, as well as the development of chemical complexes with the silicate structures of K, KNs, and KNTs [36,41]. Physically encapsulating OXAP molecules above an outer layer of chemically encapsulating drug molecules may lead to the operation of both chemical and physical processes [42].

##### Isotherm Properties

Classic Isotherm Models

The equilibrium characteristics of the OXAP-loading reactions into K, KNs, and KNTs as potential carriers have been illustrated using the conventional assumptions of Langmuir (Equation (3), Freundlich (Equation (4), and Dubinin–Radushkevich (D–R) (Equation (5). This had been completed by non-linearly fitting the results with the models’ illustrative equations while considering the correlation coefficient (R^2^) and chi-squared (χ^2^) values as indicators of the fitting degree (Table 1, Figure 6D–F).

(3) $$Q_{e}=\frac{Q_{max}\;bC_{e}}{\left(1+bC_{e}\right)}\;$$

(4) $$Q_{e}=K_{f}C_{e}^{1/n}$$

(5) $$Q_{e}=Q_{m}e^{-\beta \varepsilon^{2}}$$

The encapsulation of OXAP into K, KNs, and KNTs displays the equilibrium behaviors of the Langmuir isotherm instead of the Freundlich hypothesis in accordance with the established values of the model-fitting parameters. As a result, the OXAP molecules were uniformly encapsulated on the exterior surfaces of K, KNs, and KNTs in monolayer forms by numerous homogenously dispersed active receptors [5,39]. Additionally, the values of the RL parameter being less than one reveal the favorable encapsulation of OXAP ions in raw kaolinite as well as KNs and KNTs carriers. Additionally, the theoretical maximal OXAP encapsulation capacities of K, KNs, and KNTs were estimated as mathematical parameters of the Langmuir isotherm to be 29.6 mg/g, 309.3 mg/g, and 473.8 mg/g using the Langmuir fitting parameters.

Regarding the studied D–R model, its isotherm characteristics might significantly reveal the energetic heterogeneity of K, KNs, and KNTs as carriers of OXAP, whether they have homogeneous or heterogeneous surfaces [43]. Determining the Gaussian energy (E) as an attained theoretical parameter of the D–R model considerably emphasizes the nature of the predominant loading mechanisms, whether they have chemical or physical characteristics. While the chemical loading system displays values >16 kJ/mol, the physical loading reaction shows a Gaussian energy of less than 8 kJ/mol. Gaussian energy levels between 8 and 16 kJ/mol are indicative of complicated systems or weak chemical loading processes [5,43]. The OXAP-encapsulation processes by K, KNs, and KNTs have corresponding Gaussian energies of 5.84 kJ/mol, 4.65 kJ/mol, and 8.04 kJ/mol, respectively (Table 1). While the determined E values of K and KNs are within the same range and suggest dominant impact for the physical mechanisms during their loading with the OXAP drug, the recognized value for the loading of OXAP into KNTs displays significant effect for the weak chemical process during the reaction in addition to significant effect for the physical processes.

Advanced Isotherm Models

**Figure 7:**
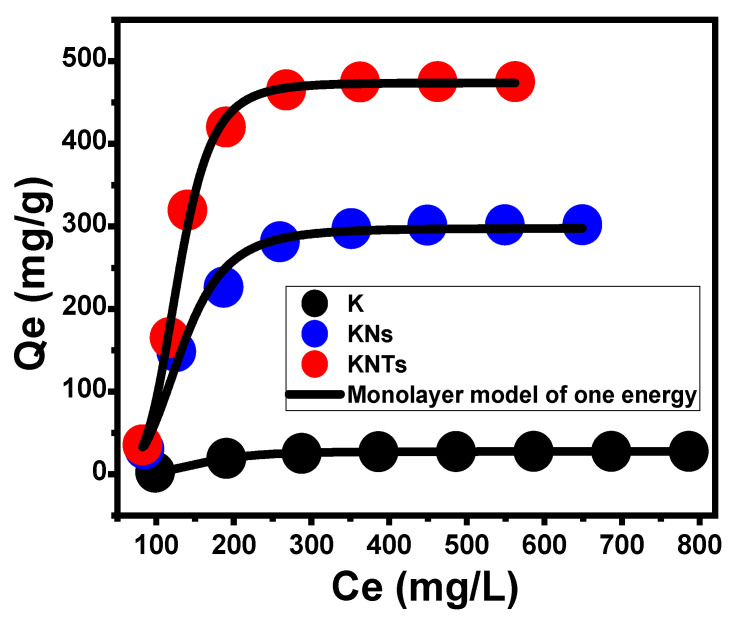
Fitting of the OXAP-loading results with the advanced monolayer model of one energy site.

The sophisticated isotherm models that were used based on the equilibrium fundamentals of statistical physics theory provide additional information about the K, KNs, and KNTs as OXAP carriers with regard to the interface between the drug in solution and the surfaces of the carriers. According to the advanced monolayer model with one energy level (Equation (6) and its mathematical parameters, either steric or energetic, the loading behaviors and the controlled mechanistic processes have been evaluated (Figure 7, Table 1). The determination coefficient (R^2^) and root mean square error (RMSE) values were considered the main determinants of the fitting degrees.

(6) $$Q={\mathrm{nN}}_{o}=\frac{{\mathrm{nN}}_{M}}{1+{\left(\frac{C1/2}{\mathrm{Ce}}\right)}^{n}}=\frac{Q_{o}}{1+{\left(\frac{C1/2}{\mathrm{Ce}}\right)}^{n}}\;$$

The model’s steric mathematical parameters included the density of occupied active receptor sites (Nm_(OXAP)_) on the surfaces of K, KNs, and KNTs, the number of OXAP molecules loaded per active site on their surfaces (n_(OXAP)_), and the OXAP-encapsulation capacities of K, KNs, and KNTs at their saturation states (Qsat_(OXAP)_). The estimated energetic parameter involved the determined encapsulation energy (∆E). The results reflected an increment in the quantities of the effective encapsulation site after the morphological transformation of kaolinite (Nm_(OXAP)_ = 6.5 mg/g) into separated nanosheets (KNs) (Nm_(OXAP)_ = 66.3 mg/g) and scrolled kaolinite nanotubes (KNTs) (Nm_(OXAP)_ = 80.86 mg/g). This strong increment in the quantities of the active sites might be attributed to the remarkable increase in the surface area and, in turn, the interaction interface, the enhancement in the reactivity of the silicate sheets, and the significant exposure of the active hydroxyl-bearing siloxane groups after the modification processes. This was reflected in the determined OXAP-loading capacities of K, KNs, and KNTs at their saturation states, which were greatly enhanced after the morphological-transformation processes from 29.9 mg/g for kaolinite to 304.9 mg/g and 473.07 mg/g for KNs and KNTs, respectively. Additionally, the detected numbers of the loaded OXAP ion per active site on the surfaces of K, KNs, and KNTs (n _(OXAP)_) demonstrate vital impact for the morphological-transformation process on their surficial properties as carriers. The estimated values of n_(OXAP)_ during the loading process of OXAP into K, KNs, and KNTs are 4.6, 4.7, and 5.85. These values are higher than 1, which impels the vertical loading of the OXAP ions on the surfaces of K, KNs, and KNTs in addition to the retention of the drug ions by multi-molecular mechanisms [44,45]. However, each active site on the surfaces of K and KNs can be loaded with about five molecules of OXAP, and each active site on the surface of KNTs can be loaded with up to six molecules of OXAP.

The loading energies (E) of OXAP into K, KNs, and KNTs were calculated using Equation (7), based on the theoretically obtained remaining OXAP concentrations at their half-saturation states (C1/2) as well as the drug’s solubility at different temperatures (Table 1).

(7) $$\Delta E=-RT\;ln\left(\frac{S}{C_{1/2}}\right)\;$$

The determined encapsulation energies of OXAP into K, KNs, and KNTs are −5.3 KJ/mol, −7.5 KJ/mol, and −4.2 KJ/mol, respectively. These values support the previous findings about the physical encapsulation mechanisms (ΔE ≤ 40 kJ/mol) of OXAP into K, KNs, and KNTs [44]. These processes might involve van der Waals forces (ΔE = 4 to 10 kJ/mol), dipole forces (ΔE = 2 to 29 kJ/mol), and hydrogen bonding (ΔE < 30 kJ/mol) [46,47].

##### Thermodynamic Properties

**Figure 8:**
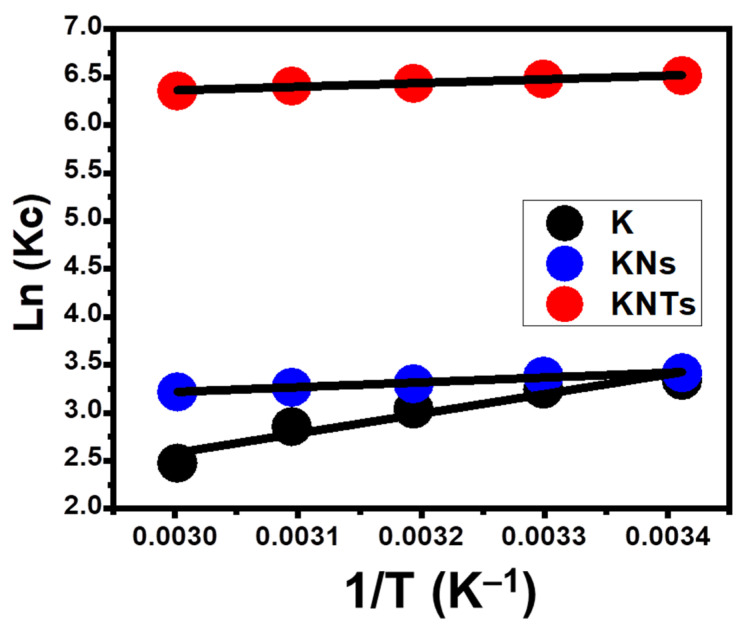
Fitting of the OXAP-loading results into K, KNs, and KNTs with Van’t Hof thermodynamic equation.

Within an operating temperature range of 20 °C to 60 °C, the thermodynamic characteristics of the OXAP-encapsulation processes by K, KNs, and KNTs were investigated. This was conducted considering the other study factors at specific values (25 mg (dosage), 24 h (duration), 50 mL (volume), 800 mg/L (OXAP concentration), and pH 8). This included the basic thermodynamic functions such as Gibbs free energy (Go) (Equation (8) as well as the entropy (ΔS°) and enthalpy (ΔH°) which were obtained by fitting the data with the Van’t Hof equation (Equation (9) (Figure 8) [27].

(8) $$\mathrm{In}\;\left(K_{c}\right)=\frac{\Delta S^{o}}{R}-\frac{\Delta H^{o}}{RT}\;$$

(9) $$\Delta G^{0}=-RT\;In\;K_{c}\;$$

Recognizing the values of ΔS° and ΔH° with negative signs demonstrates the exothermic, spontaneous, and favorable characteristics of the OXAP-encapsulation mechanisms using K, KNs, and KNTs as potential carriers. Additionally, the positively signed ΔS° values of the K, KNs, and KNTs loading systems for OXAP indicated an increase in the randomness of the reactions that occurred with regard to the temperature that was being tested.

### 2.3. In Vitro Release Profiles

**Figure 9:**
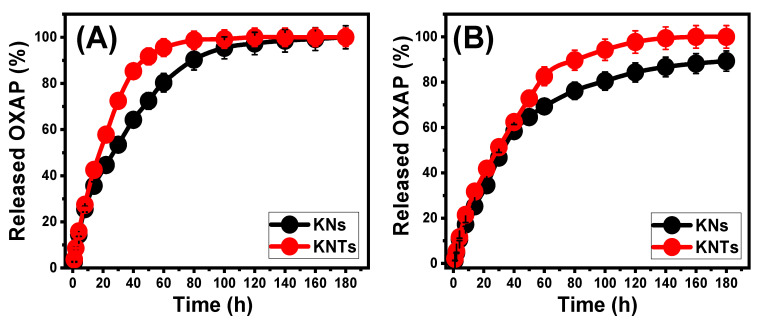
The OXAP-release profiles of KNs and KNTs either in the acetate buffer (**A**) or the phosphate buffer (**B**) (*p* < 0.005; *n* = 3).

The percentages of OXAP molecules that diffused into the two examined buffer solutions (phosphate (pH 7.4) and acetate (pH 5.5)) which acted as media to simulate the investigated tumor cells, were determined to monitor the release profiles of KNs and KNTs (Figure 9). The observed OXAP-release patterns from KNs and KNTs into the assessed buffers display significant variations in the diffusion rates as a consequence of the extension of the release period. The measured release parentages corroborate quick diffusion rates throughout the early diffusion periods of the tests, which slowly decrease until the equilibrium interval with a constant rate of diffusion or the full release of the trapped OXAP dosage (Figure 9). The notable quick diffusion of OXAP out of KNs and KNTs structures during the initial phases of accomplished tests was primarily attributed to the desorption processes of the physically adsorbed drug molecules or the weakly bonded ions with the surficial active groups of KNs and KNTs [18,48]. Following that, all of the barely loaded OXAP ions had been entirely diffused, and the release mechanisms became restricted mainly to the chemically complexed drug ions with the active groups KNs and KNTs, resulting in a decrease in release rates [12,27]. In comparison to the estimated release % in the buffered phosphate solution (pH 7.4), the established OXAP-release behavior in the tested acetate buffering solution (pH 5.5) is higher (Figure 9). Such release characteristics were attributed to the excellent mobility and solubility of OXAP in low pH solutions [15].

OXAP is typically released from KNs over a period of 180 h and 160 h, respectively, in phosphate and acetate buffers as well, without the entire diffusion state being marked. The realized maximum diffusing percentages are 100% (180 h; pH 5.5) and 89.3% (180 h; pH 7.4) (Figure 9A). Nearly 50% of the loaded dosage of OXAP diffused within 30 h (pH 5.5) and 40 h (pH 7.4). In comparison to KNs, loaded OXAP molecules inside the framework of KNTs display greater diffusion properties. After 22 h (pH 5.5) and 30 h (pH 7.4), about 50% of the loaded dosage of OXAP diffused. After 120 h (pH 5.5) and 160 h (pH 7.4), the entire diffusion state had been achieved (Figure 9B). Experiments have shown that OXAP moves more easily through KNTs than through KNs. This could be because large amounts of the drug are thought to be trapped in the pores of KNTs, and there are also many active sites that act as hubs for the weak physical loading of OXAP. Moreover, the high numbers of loaded OXAP per active site (n_(OXAP)_ = 5.85) on the surface of KNTs suggested increasing the aggregation properties of the drug molecules on the surface of KNTs as compared to KNs (n_(OXAP)_ = 4.7), which induces the release rate of the loaded dosages of the drug.

Supplying OXAP molecules directly into patients’ bodies as anticancer chemotherapy at continuous and very slow rates is encouraged during the course of therapy of the tumor cells since this method provides long-term contact and actual interaction between the cancerous cells and the introduced therapy [7,12]. In certain circumstances, it is advised to introduce the recommended therapeutic dose of the medicine at specific time intervals through the quick delivery of the medication at an abrupt rate and within short periods of time. As a result, the established delivery systems of KNs and KNTs are very successful in loading and releasing OXAP molecules at regulated levels.

### 2.4. Release Kinetic Studies

The kinetic features of the OXAP-releasing reactions from KNs and KNTs can, potentially, be used as evidence of the mechanism that operates during the diffusion reactions. The explored kinetic releasing models include the zero-order (Z-O) (Equation (10), first-order (F-O) (Equation (11), Higuchi (H-G) (Equation (12), Hixson–Crowell (H–C) (Equation (13), and Korsmeyer–Peppas (K–P) (Equation (14) models [12]. These models were assessed based on the findings of the linear regression-fitting procedures of the release results and their mathematical equations within the two buffers under study.

(10) $$W_{t}-W_{0}=K_{0}.t\;$$

(11) $$\ln\left(W_{\infty }/W_{t}\right)=K_{1}.t$$

(12) $$W_{t}=K_{h}t^{1/2}\;$$

(13) $$W_{o}^{1/3}-W_{t}^{1/3}=K_{HC}t\;$$

(14) $$W_{t}/\;W_{\infty \;}=K_{p\;}t^{n}\;$$

Based on the Z-O model assumptions, OXAP release may occur without an important impact of the loaded dose or amount on the release profiles, and the system suggests a stable diffusion rate [13]. According to the F-O supposition, the release characteristics of OXAP are significantly dependent on the loaded number of its molecules [1]. For the kinetic characteristics that match the hypothesis of Higuchi kinetics, the OXAP release occurs by means of diffusion mechanisms that are also dependent on certain parameters [1,49]. These include the following: (1) loaded OXAP diffuses at stable rates and in only one direction; (2) the quantity of the loaded dosage is greater than its actual release level; (3) the solubility and swelling characteristics of the utilized carrier have a neglected impact on the release efficiency; and (4) the addressed carrier has been distinguished by its sink nature [13]. Hixson–Crowell kinetics has been utilized to demonstrate release processes that involve the performance of erosion mechanisms and display a controlling influence of both the surface area and grain diameter of the incorporated solid carriers [13,50]. The Korsmeyer–Peppas model primarily demonstrates release systems that incorporate the collaboration of diffusion and erosion processes, particularly for the hybrid delivery system [1,51].

**Figure 10:**
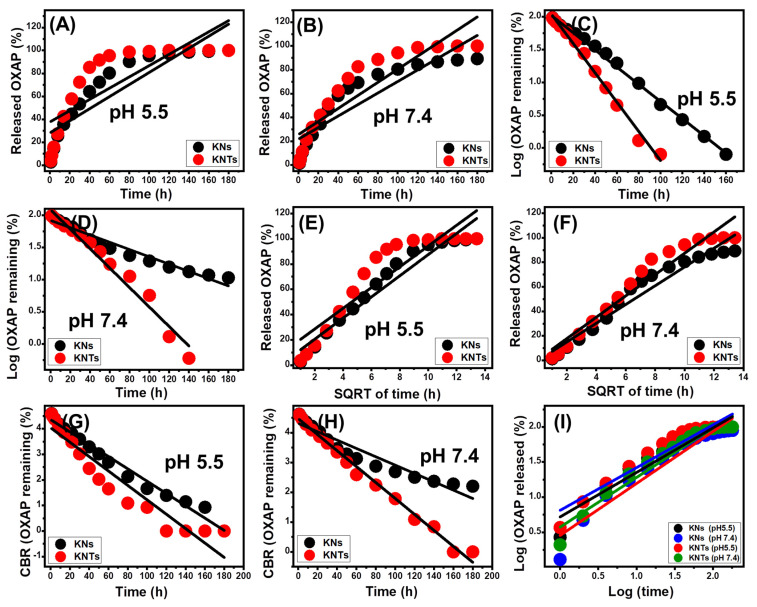
Fitting of the OXAP-release results with zero-order model (**A**,**B**), fitting of the OXAP-release results with first-order model (**C**,**D**), fitting of the OXAP-release results with Higuchi model (**E**,**F**), fitting of the OXAP-release results with Hixson–Crowell model (**G**,**H**), and fitting of the OXAP-release results with Korsmeyer–Peppas model (**I**).

Based on the calculated determination coefficient, the estimated fitting degrees show that the OXAP release data from KNs and KNTs and the kinetic characteristics of the F-O model (Figure 10C,D, Table 1) match up better than those of the Z-O model (Figure 10A,B, Table 1). Based on this kinetic behavior, the total quantity of loaded OXAP medication has a major impact on the release characteristics of both KNs and KNTs. The release findings for KNs and KNTs are in strong agreement with both Higuchi’s (Figure 10E,F; Table 1) and Hixson–Crowell’s (Figure 10G,H, Table 1) kinetic assumptions. Therefore, a combination of diffusion and erosion mechanisms were engaged in the OXAP-release behaviors of KNs and KNTs. The erosion behavior may be attributed to the partial disintegration of silicate materials at higher pH levels. The excellent match between the OXAP release behaviors and the kinetic hypothesis proposed by the Korsmeyer–Peppas model reveals the existence of diffusion processes as the major mechanism, together with the accelerating influence of erosion processes, particularly in phosphate buffers (Figure 10I, Table 1). The estimated values of the diffusion exponent (n) as fitting parameters for either KNs (0.62 (acetate) and 0.72 (phosphate) or KNTs (0.6 (acetate) and 0.7 (phosphate) verify non-Fickian transportation behaviors that are in accordance with the earlier kinetic studies about the cooperation of both diffusion and erosion mechanisms [22].

### 2.5. Cytotoxicity Properties

Colorectal cancer (HCT-116) cell lines were used to test the cytotoxicity of free KNs, KNTs, and their OXAP-loaded derivatives as potential anticancer agents and as promising carriers of enhanced biologic effect on the therapeutic properties of the loaded OXAP drug. Regarding the synthetic KNs and KNTs as free particles or unloaded materials, they produce significant cytotoxic effects, particularly at high concentrations (>50 µg/mL), on the evaluated HCT-116 tumor cell lines.

**Figure 11:**
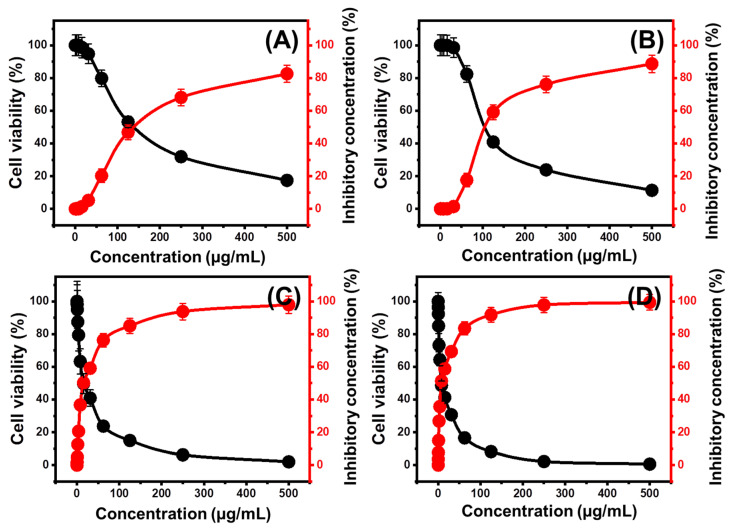
The cytotoxicity effect of free KNs (**A**), free KNTs (**B**), OXAP-loaded KNs (**C**), and OXAP-loaded KNTs (**D**) on colorectal cancer cells (HCT-116) (*p* < 0.002; *n* =3).

The free KNs (500 µg/mL) produced an inhibitory percentage of 17.41%, an IC-50 of 143.6 µg/mL, and cell viability of 82.59% (Figure 11A). The values that were determined for free KNTs are 114.3 µg/mL (IC-50), 88.68% (inhibitory percentage), and 11.32% (cell viability) (Figure 11A,B). Such significant cytotoxic qualities might be attributed to the significant surface reactivity of the separated kaolinite sheets and their scrolled products as nanotubes, in conjunction with the confirmed oxidation effects of clay nanomaterials as a result of their structural impurities of various transitional metals. Also, the results declared a remarkable impact of the one-dimensional morphology of kaolinite on its cytotoxicity as an anticancer therapy.

In regards to the cytotoxic effect of OXAP-encapsulated KNs and KNTs, they have a greater effect than OXAP alone. The determined cell viability, inhibitory percentage, and IC-50 during the incorporation of the OXAP drug as a free drug without carriers are 11.62 %, 88.38 %, and 17.85 µg/mL, respectively. OXAP-encapsulated KNs (500 µg/mL) possessed 2.04% cell viability, 97.96% inhibitory percentage, and an IC-50 of 15.40 µg/mL, respectively (Figure 11C). OXAP-encapsulated KNTs have a cell viability of 0.61%, an inhibitory percentage of 99.39%, and an IC50 of 4.53 µg/mL (Figure 11D). The applications of such carriers significantly increase the interaction interface between the cancer cells and the drug molecules, preserving prolonged and continuous interaction effects.

### 2.6. Comparison study

The saturation-loading capacities of K, KNs, and KNTs were compared to other investigated delivery systems in the literature (Table 2). The presented values declared the significantly higher OXAP-loading properties of KNs and KNTs as compared to the reported natural zeolite (philipsite), synthetic zeolite (zeolite-A), and diatomite, as well as the synthetic composites based on them. This signifies the value of the synthetic structure as an enhanced OXAP-loading system with promising loading capacities as compared to the recently evaluated structures.

## 3. Experimental Work

### 3.1. Materials

The used kaolinite powder during the preparation of kaolinite single sheets and kaolinite nanoscrolls was obtained as a refined sample from the Central Metallurgical and Development Institute in Egypt. Dimethyl sulfoxide (DMSO) (>99.5%; CAS: 67-68-5; Sigma-Aldrich), cetyltrimethylammonium bromide (CTAB) (>98%; CAS: 57-09-0; Sigma-Aldrich, St. Louis, MO, USA), and methanol (>99.9%; CAS: 67-56-1; Sigma-Aldrich) were applied during the exfoliation and scrolling steps. Oxaliplatin drug (Sigma-Aldrich; MW 397.29) [SP-4-2-(1R-trans)]-(1, 2-Cyclohexanediamine-N,N′) [ethanedioata(2--)-O,O′]platinum) was used during the loading and release tests.

### 3.2. Synthesis of Kaolinite Nanosheets (KNs) and Nanotubes (KNTs)

The kaolinite-layered units were exfoliated using a simple chemical expansion process. The raw mineral kaolinite was pulverized for 6 h in a ball mill to attain kaolinite powder with a size range of 20 to 100 µm. The pulverized mineral kaolinite (15 g) was then homogeneously mixed with 50 mL of a dilute solution of DMSO (8 (DMSO):1 (distilled water)) for 5 h by using a conventional magnetic stirring device. This process is essential for destroying the existing hydrogen bonds that link the layered silicate units of kaolinite. The subsequently formed DMSO-treated kaolinite had been washed using methanol for 20 min; this procedure was performed five times to remove the intercalated DMSO molecules and replace them with the alcohol molecules, forming an organophilic product known as methoxy kaolinite (Mth/K). The obtained Mth/K particulates were homogenized with a previously prepared CTAB solution (20 g CTAB + 50 mL distilled water) for 48 h via a complex mixing system composed of a magnetic stirrer in addition to an ultrasound source (240 W), which results in the formation of exfoliated or separated kaolinite nanosheets (KNs). Following that, the resultant KNs particulates were thoroughly rinsed with distilled water, then slowly dried at 65 °C over 12 h, and then named KNs.

After the kaolinite sheets were successfully peeled off, the system was given more CTAB (15 g), and the resulting mixture was sonicated for an extra 48 h at 80% power (240 W) to ensure that the ductile silicate sheets of kaolinite had grown and been rolled into nanoscrolls or nanotubes. The end-product was then separated from the mixture, washed with a mixture of methanol and distilled water, and dried at 65 °C for 10 h.

### 3.3. Analytical Techniques

Using a PANalytical-Empyrean X-ray diffractometer within a measurement range of 0 to 70°, the degree of crystallinity and crystalline phases were detected according to the obtained XRD patterns. The chemical groups of KNs and KNTs as well as the synthetic intermediate compounds were determined by a Fourier-transform infrared spectrometer (FTIR8400S; Shimadzu, Kyoto, Japan) within detection ranges of 400 cm^−1^ to 4000 cm^−1^. The expected changes in the morphological properties of kaolinite during the different modification procedures were verified based on the SEM images, which were captured using a scanning electron microscope (Gemini, Zeiss Ultra 55) immediately after coating the modified clay particles with thin films of gold. Furthermore, the interior features of scrolled and exfoliated kaolinite were evaluated depending on their HRTEM images, which were obtained by a transmission electron microscope (JEOL-JEM2100, Tokyo, Japan) at a 200 kV accelerating voltage. On the basis of the corresponding N_2_ adsorption/desorption isotherms, both the surface area and porosity of KNs and KNTs were measured with a surface-area analyzer (SA3100, Beckman Coulter Co.; USA).

### 3.4. OXAP Loading Studies

The encapsulating capacities of K as well as synthetic KNs and KNTs as delivery structures for OXAP were assessed under the influence of several variables to regulate the loading capacity. The loading pH (2–9), loading duration (1–24 h), tested OXAP concentration (100–800 mg/L), and experimental temperature (20–60 °C) were all examined as the controlling loading parameters. A vortex rotator device was used during the loading processes to homogenize carrier particles in 50 mL of OXAP solutions after taking into consideration all other variables that influence the reactions. After the completion of each experiment, the utilized particulates of K, KNs, and KNTs were extracted from the aqueous solutions of the OXAP drug via Whatman filter paper. The residual OXAP molecules in the filtrates were measured by UV–Vis spectrophotometer (λ_(max)_ = 209 nm), and the determined concentrations were applied to calculate the quantities that were loaded in mg/g using Equation (15). All loading assays were completed in three separate experiments, and the displayed OXAP concentrations were the averages of all three experiments.

(15) $$\mathrm{Loaded}\;\mathrm{drug}\;\left(\mathrm{mg}/g\right)=\frac{\left(\mathrm{Initial}\;\mathrm{concentration}-\mathrm{Residual}\;\mathrm{concentration}\right)\times \;\mathrm{solvent}\;\mathrm{volume}}{\mathrm{Carrier}\;\mathrm{weight}}\;$$

### 3.5. The Release Studies

The release properties and diffusion patterns of OXAP from the investigated K, KNs, and KNTs carriers were studied in two releasing media with different pH values (phosphate-buffered saline solution, pH 7.4, and acetate-buffered saline solution, pH 5.5) and a fixed temperature of 37.5 °C. The two independent tests for the release processes entailed homogenizing the loaded K, KNs, and KNTs carriers (100 mg/g of OXAP) throughout 500 mL of each of the mentioned buffers. The homogenization procedure was carried out using a DISTEK dissolution device for 180 h as the entire release duration at a fixed rotation speed of 200 rpm. The actual concentrations of the released OXAP were measured using a UV–Vis spectrophotometer (λ_(max)_ = 209 nm) based on regularly extracted samples (5 mL) from both of the buffers that were used. These extracted samples were subsequently reinserted into the overall release buffers to verify that the process occurred under identical conditions. This was repeated three times, and the mean results were employed to calculate the release percentages using Equation (16).

(16) $$\mathrm{Drug}\;\mathrm{release}\;\left(\%\right)=\frac{\mathrm{Amount}\;\mathrm{of}\;\mathrm{Released}\;\mathrm{OXAP}\;}{\mathrm{Amount}\;\mathrm{of}\;\mathrm{loaded}\;\mathrm{OXAP}}\times 100\;$$

### 3.6. In Vitro Cytotoxicity

#### 3.6.1. Cell Lines

Colorectal cancer cell lines (HCT-116) from the American Type Culture Collection (ATCC, Rockville, MD, USA) were used during the cytotoxic assays. Chemical and biological reagents, such as 0.25% trypsin-EDTA, dimethyl sulfoxide (DMSO), gentamycin, 3(4,5-dimethylthiazol-2-yl)-2.5 diphenyltetrazolium bromide (MTT 99%), fetal bovine serum, DMEM, HEPES buffer, and RPMI-1640, were used during the incubation and cytotoxic assay. All the included cytotoxicity tests were completed at the Regional Center for Mycology and Biotechnology, Al-Azhar University, Egypt.

#### 3.6.2. In Vitro Cytotoxicity

First, the selected HCT-116 cell lines were cultivated under very strict conditions in RPMI-1640 medium with 10% fetal calf serum and 50 µg/mL gentamycin at 37 °C and 5% carbon dioxide. The cancerous cell lines (5 × 10^4^ cells/well) were then immersed in Corning^®^ 96-well plates over 24 h after the three-times-per-week culture process. Then, certain quantities of the OXAP-loaded K, KNs, and KNTs carriers were added to all of the cell strains, and they were cultured a second time for a further 24 h. Using the widely used MTT cell-proliferation assay technique, the number of viable cells generated throughout the duration of incubation was determined. By fulfilling the incubation stage, the cultivation media that had been incorporated had been successfully eliminated and replaced with newly produced media (100 µL of RPMI). The freshly added media was carefully blended together with the MTT (10 µL; 12 mM) and cultivated once more for 5 h until the remarkable formation of formazan that had a distinctive purple color. The formazan was then successfully dissolved with 50 µL of DMSO solution. The final stage involved measuring the optical density of the cell lines that had been cultivated throughout the experiments using a microplate at a measurement wavelength of 590 nm. The values determined were applied to calculate cell viability% using Equation (17) [5].

(17) $$\mathrm{Cell}\;\mathrm{viability}\;\left(\%\right)=\frac{\mathrm{Mean}\;\mathrm{OD}}{\mathrm{Control}\;\mathrm{OD}\;}\times 100\;$$

## 4. Conclusions

Chemical exfoliation and scrolling of kaolinite in single sheets (KNs) and nanotubes (KNTs), respectively, were evaluated as advanced modification methods to obtain highly reactive modified forms of kaolinite with enhanced properties as drug-delivery systems for oxaliplatin. The synthetic modified forms (KNs and KNTs) showed significant enhancement in the OXAP-loading properties (304.9 mg/g for KNs and 473 mg/g for KNTs). This was assigned experimentally to the strong increase in the surface area, surface reactivity, and exposure of the active sites. Theoretically, this was attributed mainly to the increase in the quantities of the active loading sites (80.8 mg/g (KNTs) and 66.3 mg/g (KNs)) as compared to kaolinite (6.5 mg/g) as well as the number of loaded OXAP ions per site. These results also illustrate the higher loading properties of KNTs as compared to KNs. The loading reactions occurred mainly by multimolecular physical mechanisms in which the loaded ions were loaded in vertical form based on the Gaussian energies (<8 KJ/mol) and loading energies (<40 KJ/mol). The advanced forms of kaolinite as carriers have prolonged and continuous release profiles that consume about 100 h, which is enhanced behavior as compared to the profile of kaolinite. The release properties occurred according to non-Fickian transport characteristics with complex diffusion and erosion mechanisms. Concerning their cytotoxic impacts on HCT-116 cancer cell lines, free KNs and KNTs had cell viability percentages of 71.4% and 11.32%, respectively, whereas the OXAP-loaded samples had cell viability percentages of 2.04% (KNs) and 0.61% (KNTs). These supported the obtained forms of modified kaolinite to be applied as delivery systems for the OXAP drug, which can be confirmed by further in vivo studies considering the different biological aspects.

## Figures and Tables

**Table 1:** The obtained mathematical parameters of the studied kinetic, classic isotherm, advanced isotherm, thermodynamic, and release kinetic models.

Model	Parameters		K	KNs	KNTs
Kinetic models					
Pseudo-first-order	K_1_ (min^−1^)		0.175	0.155	0.253
	Qe_(Cal)_ (mg/g)		21.47	166.6	250.7
	R^2^		0.94	0.94	0.90
	X^2^		0.52	4.1	5.73
Pseudo-second-order	k_2_ (g mg^−1^ min^−1^)		0.0046	4.9 × 10^−4^	6.51 × 10^−4^
	Qe_(Cal)_ (mg/g)		29.44	234.5	334.2
	R^2^		0.91	0.92	0.85
	X^2^		0.73	5.4	7.3
Isotherm models					
Langmuir	Q_max_ _(mg/g)_		29.63	309.3	473.8
	b (L/mg)		0.001	0.0057	6.93 × 10^−4^
	R^2^		0.99	0.99	0.99
	X^2^		0.03	3.67	0.55
	RL		0.55–0.90	0.18–0.63	0.64–0.93
Freundlich	1/n		0.155	0.33	0.92
	k_F_ (mg/g)		0.8	6.4	1.69
	R^2^		0.99	0.99	0.99
	X^2^		0.08	4.2	2.11
D–R model	β (mol^2^/KJ^2^)		0.0146	0.0231	0.0077
	Qm (mg/g)		30.8	337.13	550.2
	R^2^		0.99	0.98	0.96
	X^2^		0.148	1.64	7.6
	E (KJ/mol)		5.84	4.65	8.04
Monolayer model of one energy	n		4.6	4.7	5.85
	Nm (mg/g)		6.5	66.3	80.86
	Q_(sat)_ (mg/g)		29.9	304.9	473.07
	∆E (kJ/mol)		−5.3	−7.5	−4.2
Thermodynamics					
∆G° (kJ mol^−1^)	293.13		−8.13	−8.32	−15.88
	303.13		−8.18	−8.52	−16.33
	313.13		−7.90	−8.60	−16.75
	323.13		−7.67	−8.77	−17.21
	333.13		−6.85	−8.91	−17.60
ΔH° (kJ mol^−1^)				−4.16	−3.88
ΔS° (J K^−1^ mol^−1^)				14.24	18.7
Release kinetics					
Models	KNs		Determination coefficient		
			KNTs		
	Acetate buffer (pH 5.5)	Phosphate buffer (pH 7.4)	Acetate buffer (pH 5.5)		Phosphate buffer (pH 7.4)
Zero-order	0.75	0.79	0.61		0.78
First-order	0.99	0.94	0.99		0.97
Higuchi	0.92	0.93	0.89		0.94
Hixson–Crowell	0.97	0.91	0.90		0.99
Korsmeyer–Peppas	0.94	0.93	0.93		0.91
n	0.62	0.72	0.60		0.70

**Table 2:** Comparison between the loading capacities and release periods of the studied carrier and other carriers in literature.

Carrier	Loading Capacity (mg/g)	References
Hydroxyapatite	49.1	[52]
Zeolite-A	109.03	[53]
Cellulose/zeolite-A	285.7	[4]
Phillipsite	79.6	[54]
Β-cyclodextrin/phillipsite	291.5	[55]
Diatomite	65.9	[56]
Β-cyclodextrin/diatomite	238.7	[57]
kaolinite	29.9	This study
KNs	304.9	This study
KNTs	473.07	This study

## Data Availability

Data are available upon reasonable, by the corresponding authors.

## Supplementary Materials

The following supporting information can be downloaded at: https://www.mdpi.com/article/10.3390/molecules28135158/s1. Figure S1. SEM image (A) and HRTEM image (B) of the used raw kaolinite. Figure S2. FT-IR spectra of OXAP loaded raw kaolinite (A), exfoliated kaolinite sheets (B), and the synthetic kanolinite nanotubes (C).
