# Supplementary material for: Insight into the Morphological Properties of Nano-Kaolinite (Nanoscrolls and Nanosheets) on Its Qualification as Delivery Structure of Oxaliplatin: Loading, Release, and Kinetic Studies
Source: Molecules. 2023 Jul 1;28(13):5158. doi: 10.3390/molecules28135158 (PMC10343709; doi:10.3390/molecules28135158)
Supplement: Supplementary file 1 [file molecules-28-05158-s001.zip › molecules-2447116-supplementary.pdf]

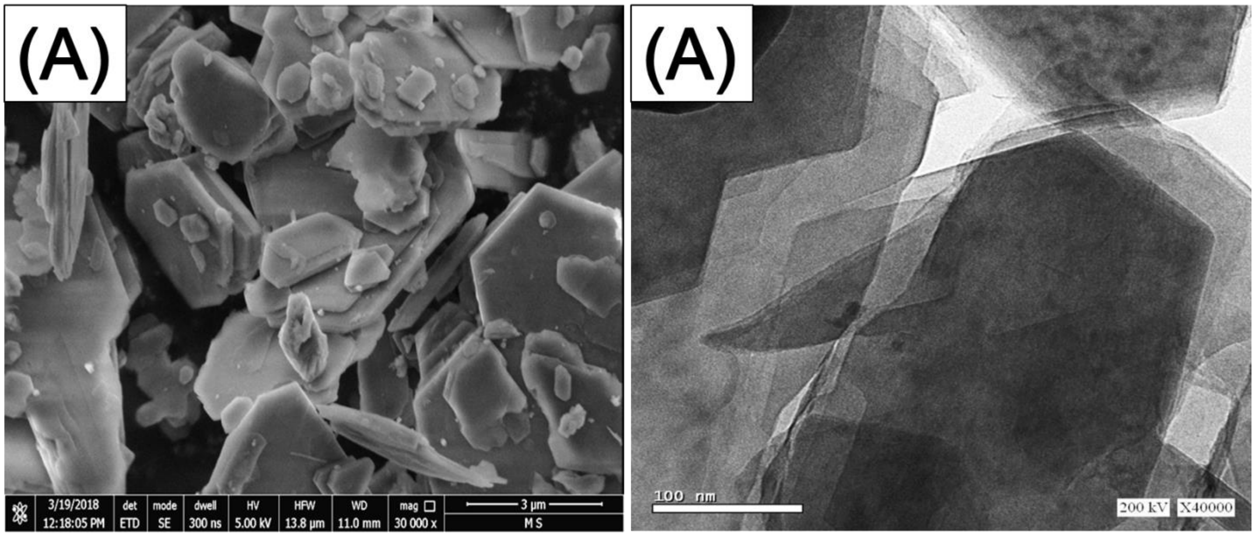

Figure S1. SEM image (**A**) and HRTEM image (**B**) of the used raw kaolinite.

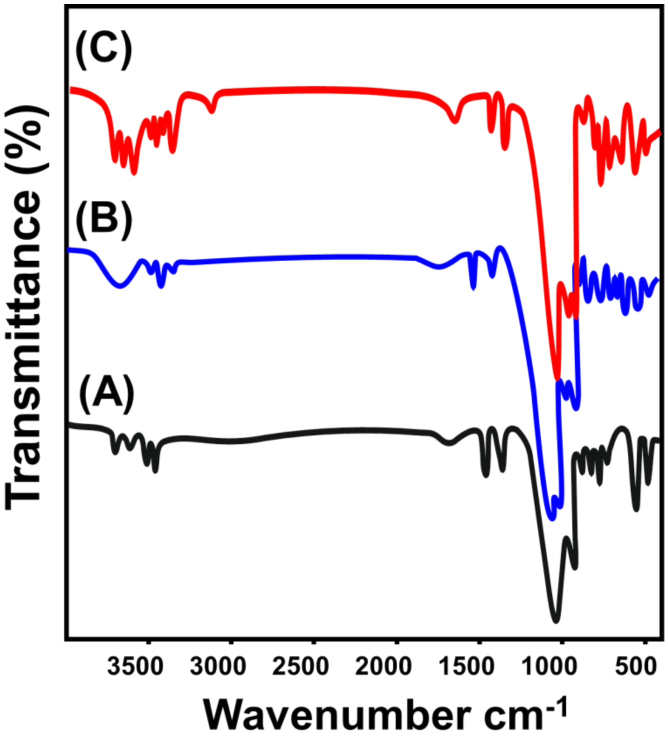

Figure S2. FT-IR spectra of OXAP loaded raw kaolinite (**A**), exfoliated kaolinite sheets (**B**), and the synthetic kanolinite nanotubes (**C**).
